# Genome‐Wide Detection of Copy Number Variation and Association Studies With Physiological and Anatomical Indicators of Heat Stress Response in Lactating Sows

**DOI:** 10.1111/jbg.70009

**Published:** 2025-08-12

**Authors:** Letícia Fernanda de Oliveira, Renata Veroneze, Lorena Ferreira Benfica, André Campelo Araujo, Yijian Huang, Jay S. Johnson, Luiz F. Brito

**Affiliations:** ^1^ Department of Animal Science Federal University of Viçosa Viçosa Minas Gerais Brazil; ^2^ Department of Animal Sciences Purdue University West Lafayette Indiana USA; ^3^ Smithfield Premium Genetics Rose Hill North Carolina USA; ^4^ Division of Animal Sciences University of Missouri Columbia Missouri USA

**Keywords:** climatic resilience, landrace, large white, maternal‐line pigs, structural variants

## Abstract

Indicators of heat stress response are heritable complex traits with polygenic inheritance. Copy number variations (CNV) are important genomic structural variations that may be linked to climatic adaptation by influencing the phenotypic variability of traits related to thermal stress and disease resistance in animals. Therefore, the primary objectives of this study were to detect CNV and CNV regions (CNVR) in pigs and explore their associations with physiological and anatomical indicators of HS response in lactating sows. A total of 4184 autosomal genome CNV (4012 deletions and 172 duplications) were detected in 969 animals. CNVR were identified by merging CNV with at least 1‐bp overlap, which enabled the identification of 236 autosomal CNVR. The association analyses led to the identification of three CNVR significantly associated with ear skin temperature and one CNVR significantly associated with vaginal temperature considering all records, vaginal temperature measured at 8:00 h, and hair density. Eleven genes harboured the CNVR with significant associations. In summary, various CNV and CNVR were identified in crossbred maternal‐line pigs, including CNVR significantly associated with physiological and anatomical heat stress response indicators in lactating sows. Candidate genes involved in immune and stress responses overlapped with the significant CNVR, suggesting that they may contribute to climatic resilience in pigs. The findings of this study contribute to better understanding the genetic background of heat stress response in lactating sows.

## 1 Introduction

The rapidly increasing global temperatures are a threat to animal production systems, most notably through the increased prevalence of heat stress (HS). HS is an especial challenge for pigs due to intensive selection for increased pig performance (i.e., higher metabolic heat production) and their lack of functional sweat glands, which restricts their capacity for evaporative cooling and impairs effective thermoregulation (Ingram et al. 1973; Johnson 2018). Breeding for improved climatic resilience is a viable strategy, as there is substantial genetic variability in pigs' responses to HS (Tiezzi et al. 2020; Wen et al. 2023; Freitas et al. 2024). To effectively leverage this variability, it is essential to understand the underlying genetic mechanisms controlling traits related to HS response. While single nucleotide polymorphisms (SNPs) have been widely studied, structural variants such as copy number variations (CNV) represent another important source of genetic variability (Redon et al. 2006; Chen et al. 2012; Pierce et al. 2018; Lye and Purugganan 2019). CNV are genomic structural variations of segments of DNA that are 50 base pairs (bp) or larger and are present at a variable copy number when compared to a reference genome (Feuk et al. 2006; Alkan et al. 2011). CNV can be categorised into insertions, deletions and duplications (Feuk et al. 2006). After detecting the CNV in the individuals, CNV regions (CNVR), which are regions with overlapping or adjacent insertions or deletions in DNA across the population, can be identified (Redon et al. 2006).

CNV are sources of genetic variation that influence gene expression within and near the modified regions, altering phenotypes and generating phenotypic variability in complex traits (Henrichsen et al. 2009). The expression of genes is typically higher in regions with more duplications or repeated copies of the gene, and lower in regions with fewer CNV or deletions (Orozco et al. 2009). Furthermore, through structural changes in the genome, CNV can lead to the expansion of gene families and the creation of new genes, especially by accumulation of recent duplications in CNVR (Juan et al. 2013). Since CNV affect phenotypic variability and can be heritable when present in germline cells (Bruder et al. 2008; Piotrowski et al. 2008), their frequency and distribution within a population can be influenced by both natural and artificial selection (Hou et al. 2011; Lye and Purugganan 2019).

There are several studies suggesting that CNVR are involved in environmental adaptation by regulating traits related to stress tolerance, disease resistance and other key physiological functions (Iskow et al. 2012; Paudel et al. 2013; Wang et al. 2016; Hull et al. 2017; Lauer et al. 2018; Cayuela et al. 2021). For instance, Paudel et al. (2013) reported that CNVR could be related to adaptation in the wild and behavioural changes during pig domestication. Kumar et al. (2023) reported genes within CNVR potentially involved in biological processes related to indigenous cattle's adaptability and disease resistance. For example, CNVR were related to local adaptation to cold water, temperature and reproductive isolation in Capelin fish ( *Mallotus villosus* ) populations (Cayuela et al. 2021); to adaptation to high temperature and humidity in horses (Wang et al. 2022); and to adaptation to tropical environments, including heat stress (HS), disease resistance and feed scarcity in African cattle breeds (different breeding purposes; Wang et al. 2016). Therefore, the influence of CNV in gene functions impacts phenotypes and can potentially result in improved resilience to specific environmental challenges (Paudel et al. 2013; Wang et al. 2016; Cayuela et al. 2021).

Although there are studies investigating CNV in pigs (e.g., Chen et al. 2012; Dong et al. 2015; Stafuzza et al. 2019; Qian et al. 2023), there is a lack of studies investigating the relationship between CNV and HS resilience in pigs, particularly during lactation, which is considered a highly heat sensitive stage of pig production (Vilas Boas Ribeiro et al. 2018). Animals with greater resilience to HS may exhibit physical and physiological adaptations, such as greater skin surface area, lower metabolic heat production, adjusted blood flow and increased heat shock protein expression, that enhance thermoregulation and promote survival under high temperatures (Johnson 2018). These adaptations help the animals maintain a more stable body temperature. Understanding the genetic background of HS response in pigs and the contribution of CNV to its phenotypic variability could provide valuable insights for breeding animals with greater thermal resilience. Investigating the association of CNV with physiological and anatomical indicators of HS response could provide insights into the genetic mechanisms underlying climatic resilience in pigs. Therefore, our primary objectives were to: (1) detect CNV and CNVR in a crossbred (Large White × Landrace) pig population, and (2) investigate the potential associations between the identified CNVR and various physiological and anatomical indicators of HS response in lactating sows.

## 2 Material and Methods

### 2.1 Animals and Genotypes

The data used in this study was previously described by Johnson et al. (2023) and Freitas et al. (2023). In summary, the phenotypes were collected from 1645 multiparous lactating sows (Large White × Landrace cross) at a commercial sow farm in Maple Hill, North Carolina, USA. A total of 1639 sows were genotyped using the PorcineSNP50K Bead Chip (Illumina, San Diego, CA, USA) containing 50,703 SNPs. Quality control was performed to remove samples with poor quality and to reduce false‐positive CNV detection. First, the genotypes with GenCall scores lower than 0.15 were assumed to be missing data. SNPs were removed when they had unknown or duplicated positions in the genome or when their call rate was lower than 90%. A total of 1613 animals and 48,944 SNPs remained for subsequent analyses, including 46,580 autosomal SNPs and 2355 SNPs located on the X chromosome.

### 2.2 Phenotypes

The phenotypes used in this study and the data collection were previously described by Johnson et al. (2023) and Freitas et al. (2023). The phenotypes recorded include respiration rate (RR), ear skin temperature (T_ES_), shoulder skin temperature (T_SS_), rump skin temperature (T_RS_), tail skin temperature (T_TS_), automatically‐recorded vaginal temperatures (T_V_) and panting score (PS). Six vaginal temperature traits were derived based on automatically recorded data: T_Vall_ (measurements at each 10‐min interval), T_V4days_ (average of six records per hour at 08:00, 12:00, 16:00 and 20:00 h over four days), and single records at 08:00, 12:00, 16:00 and 20:00 h on the first day (T_V8h_, T_V12h_, T_V16h_ and T_V20h_). Furthermore, the phenotypic dataset included the following anatomical traits: hair density (HD), body condition score using a sow calliper (BCS_cal_) (Knauer and Baitinger 2015), visual body condition score (BCS_vis_), body size (BS), ear length (EL) and ear area (EA).

The phenotypes were pre‐corrected for fixed effects as described in Freitas et al. (2023). In summary, the fixed effects fitted for skin surface temperature (T_ES_, T_SS_, T_RS_ and T_TS_) and RR were trait recorder; concatenation of week, day, and time of measurement; parity; days in lactation; concatenation of barn type and room; and in‐barn environmental temperature as a linear covariate. For PS, the fixed effects were trait recorder; concatenation of week and day of measurement; parity; days in lactation; concatenation of barn type and room; and in‐barn environmental temperature as a linear covariate. For T_Vall_, the fixed effects were the concatenation of week and day of measurement; parity; concatenation of barn type and room; and in‐barn environmental temperature as a linear covariate. For T_V4days_, the fixed effects were concatenation of week, day, and time of measurement; parity; days in lactation; concatenation of barn type and room; and in‐barn environmental temperature as a linear covariate. For T_V8h_, T_V12h_, T_V16h_ and T_V20h_, the fixed effects were the concatenation of week and day of measurement; parity; days in lactation; concatenation of barn type and room; and in‐barn environmental temperature as a linear covariate. For HD, the fixed effects fitted were trait recorder and parity. For BS, the fixed effects fitted were trait recorder, week of measurement and parity. For BCScal and BCSvis, the fixed effects fitted were trait recorder, week of measurement, parity, days in lactation and concatenation of barn type and room. For EA and EL, the fixed effects fitted were trait recorder and picture quality. Traits with repeated measurements had the average of the adjusted phenotype computed and used as the response variable for the CNVR association analyses (Sahana et al. 2023).

### 2.3 CNV Detection and CNVR Identification

The CNV detection was performed for autosomal chromosomes and the X chromosome separately, as suggested by Wang et al. (2007). Individual‐based CNV detection was performed using the PennCNV software (Wang et al. 2007), which is based on a hidden Markov model and incorporates multiple sources of information to obtain accurate CNV detection. The measures used by PennCNV (Wang et al. 2007) include the distance between SNPs, log R ratio (LRR), B allele frequency (BAF) and population frequency of the B allele (PBF). LRR is a normalised intensity value, which is computed as the allelic intensity ratio between the observed normalised intensity of a subject sample and the expected intensity based on the observed allelic ratio (Peiffer et al. 2006). BAF is a measurement used in SNP array analyses to determine the allelic intensity ratio of the B allele (Peiffer et al. 2006).

The LRR and BAF were exported from Illumina GenomeStudio software (Illumina Inc., San Diego, CA, USA). PBF was calculated from the BAF value of each SNP in all samples using the ‘compile_pbf.pl’ pipeline from the PennCNV software (Wang et al. 2007). We also performed an adjustment for genomic waves using the flag*—gcmodel* in the PennCNV software (Wang et al. 2007). Genomic waves refer to a signal noise related to the guanine‐cytosine (GC) content in the genome, which can interfere in the CNV detection and may increase the number of false‐positive CNVs. The porcine *gcmodel* was generated by calculating the GC content in the porcine genome in a region of 500 Kb around each SNP and based on a regression model of LRR value on GC content (Diskin et al. 2008). After CNV calling, a sample‐based quality control was performed to reduce false‐positive CNV detection. The quality control consisted of removing CNVs with less than three consecutive SNPs, BAF drift lower than 0.01, standard deviation of LRR greater than 0.40, minimum length of 1 Kb, maximum length of 5000 Kb and GC wave factor lower than 0.10 (after genomic waves were corrected by guanine‐cytosine content).

The CNVR were determined by merging the raw CNV that overlapped by at least 1 bp within each region, using the *mergeBed* option of the BEDtools suite tool (Quinlan and Hall 2010). After the merging step, CNVRs identified in only one animal were removed, and only CNVRs identified in at least 1% of the population were considered in subsequent analyses. Although there is a possibility that CNVs exist within the loci reported in only one animal, they are rare cases (Bruder et al. 2008; Fadista et al. 2008; Piotrowski et al. 2008). Nonetheless, considering CNVRs found in two or more animals can reduce the false‐positive rate (Fadista et al. 2008). CNVRs were classified as deletions when the region exhibited a deletion in a chromosomal segment, duplications for repeated chromosomal regions, and mixed when both deletions and duplications were identified in the same genomic region for different individuals.

### 2.4 Association Analyses

The association analyses were performed considering the only CNVR identified in at least 1% of the population. To verify if the CNVR identified was associated with the pre‐adjusted phenotypes, the following model was fitted:

$$y=1\mu +x_{i}b_{i}+\mathrm{Zu}+\varepsilon$$

where y is the vector of pre‐corrected phenotypes for each analysed trait; 1 is a vector of ones; μ is the overall mean; $b_{i}$ is the fixed effect of the CNVR i, $x_{i}$ is a vector containing the genotype score for the CNVR i, which was coded as −1, 0, or 1 if the CNV on the tested region was a deletion, absent, or a duplication in the individual, respectively; u is the random vector of polygenic effect with $u\sim N\left(0,G\sigma_{u}^{2}\right)$, where G is the genomic relationship matrix computed according to the first method proposed by VanRaden (2008), $\sigma_{u}^{2}$ is the additive genomic variance of polygenic effects; Z is the incidence matrix for u; and ε is a random vector of residual effects with $\varepsilon \sim N\left(0,I\sigma_{\varepsilon }^{2}\right)$, where I is an identity matrix, and $\sigma_{\varepsilon }^{2}$ is the residual variance. The variance components were previously estimated fitting the above‐described model excluding the CNVR from the model. The variance components estimation and the CNVR effect size estimation were performed using the BLUPF90 program (Misztal et al. 2014).

The model adjusted provided the effect size ($\hat{\beta }$) and standard error (SE) for each CNVR, which were used to compute the *t* statistic ($t=\hat{\beta }/\mathrm{SE}$). Then, the *p*‐values were obtained assuming the *t*‐distribution with n−2 degrees of freedom, where n is the sample size (i.e., the number of animals used to obtain the CNVR effect for each pre‐adjusted phenotype). To assess potential stratification due to population structure, the genomic inflation factor (λ) and its 95% confidence interval were evaluated (van den Berg et al. 2019); when λ differed substantially from 1, *p*‐values were adjusted by dividing the chi‐squared statistics by λ, following the method proposed by Devlin and Roeder (1999). Due to multiple tests, a Bonferroni correction considering α = 0.05 genome‐wise significance level was applied by dividing α by the number of CNVR. As each trait was fitted individually, no adjustment for the number of traits was considered.

### 2.5 Gene Annotation and Functional Analyses

Genes and QTLs that overlapped with the CNVR significantly associated with the pre‐adjusted phenotypes were annotated. The gene annotations in these regions were performed using the GALLO R package (Fonseca et al. 2020), utilising data for *Sus scrofa* sourced from the Ensembl database (www.ensembl.org/Sus_scrofa/Info/Index). The Database for Annotation, Visualization, and Integrated Discovery (DAVID) (Huang et al. 2009; Sherman et al. 2022) was used for conducting Gene Ontology (GO) and KEGG pathway enrichment (*p* < 0.05) analyses to identify biological processes, molecular functions, cellular components and biological pathways associated with the positional candidate genes identified.

## 3 Results

### 3.1 CNV and CNVR Detection in Autosomal Chromosomes

A total of 8270 CNV were detected in autosomal chromosomes in 1485 samples. After the quality control, 4184 CNV (4012 deletions and 172 duplications) for 969 animals remained for subsequent analyses. The number of CNV in autosomal chromosomes per individual ranged from 1 to 17, with an average of 4.32 ± 2.86 CNV per individual. The minimum number of CNV per chromosome across all samples was 50, which was for chromosomes 7 and 18. On the other hand, the highest number of CNV per chromosome across all samples was observed in chromosome 15 (627 individual CNV). The length of the CNV ranged from 2646 bp to 1352,876 bp, with an average length of 170,292.4 ± 137,912.2 bp.

**FIGURE 1:**
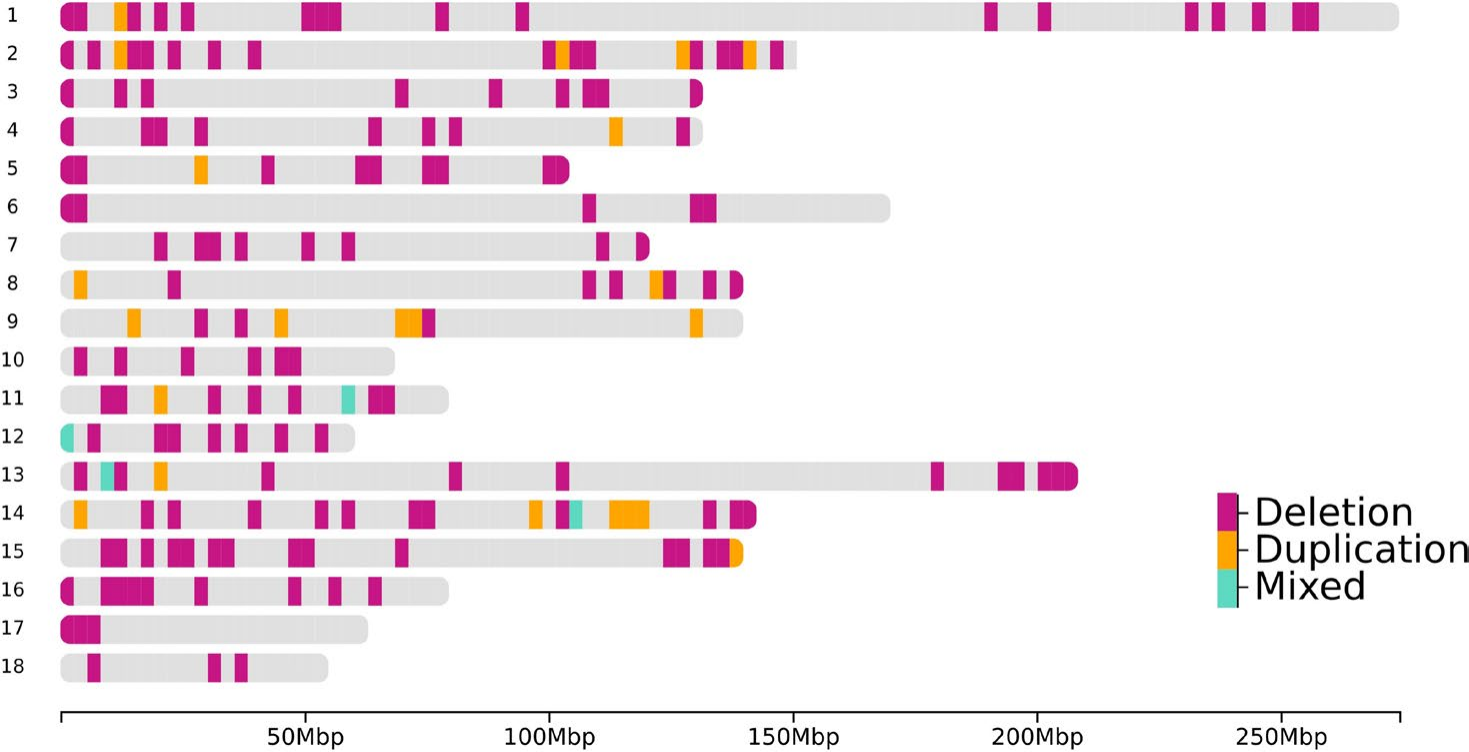
Distribution of copy number variation regions (deletion, duplication and mixed type) across autosomal chromosomes. [Colour figure can be viewed at wileyonlinelibrary.com]

**FIGURE 2:**
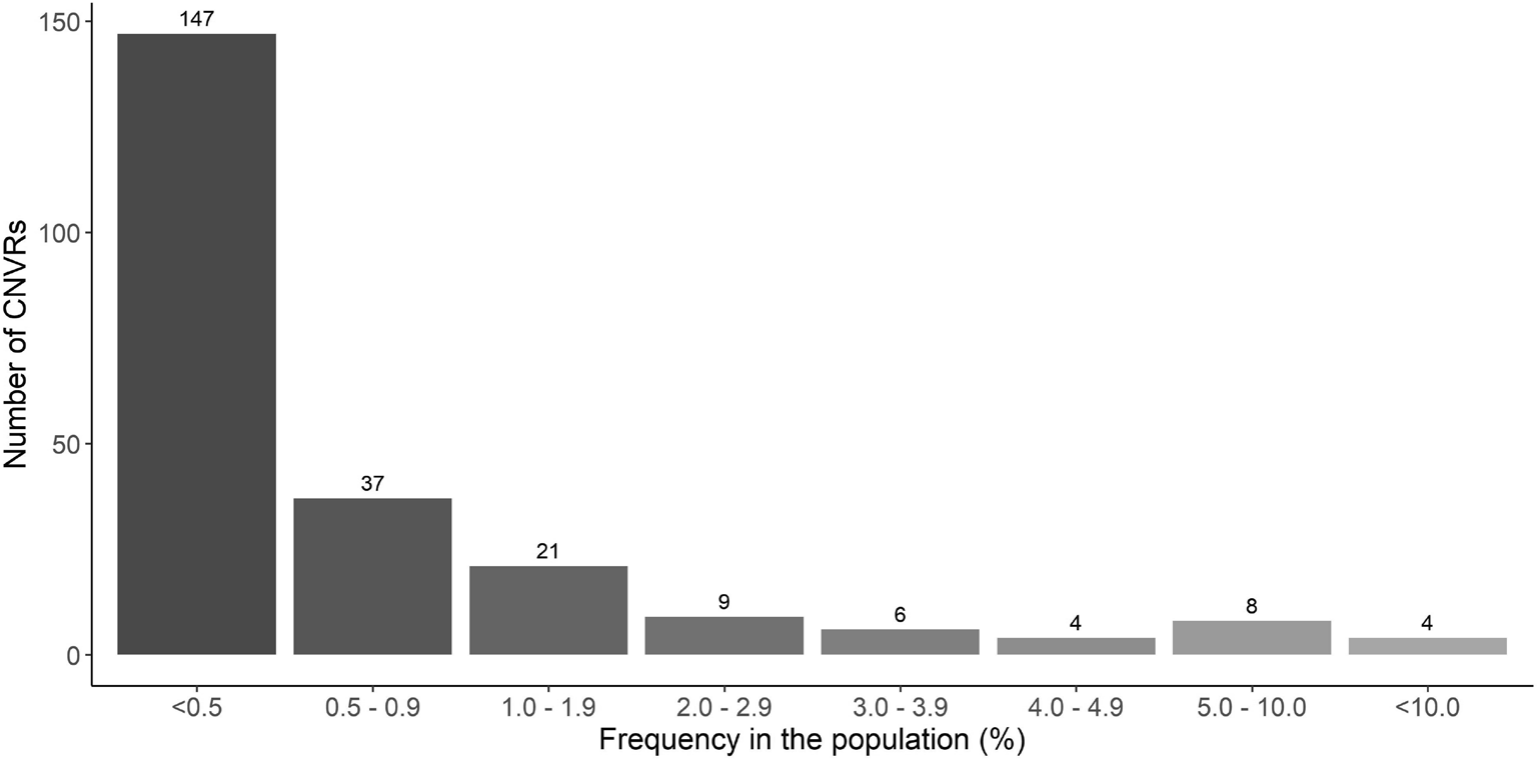
Number of copy number variation regions (CNVR) categorised based on their frequency in the population.

All 4184 CNV remaining after quality control were used to infer CNVR by merging CNV with at least 1 bp overlap. After merging, we observed 399 CNVR. However, 163 CNVR were removed from subsequent analyses due to being observed in only one sample. Thus, 236 CNVR were identified (Table S1), including 201 CNVR classified as deletion type, 30 duplication, and five mixed types (i.e., the same chromosomal segment was a deletion or duplication in different samples) (Figure 1). The length of the CNVR ranged from 14,161 bp to 1,938,601 bp with an average length of 249,698.9 ± 276,298.7 bp. The lowest number of CNVR per chromosome was observed for chromosome 18, which presented four CNVR covering 1.18% of the chromosome. Chromosome 2 had the highest number of CNVR per chromosome (*n* = 26) covering 3.56% of its length (Table 1). Chromosome 11 presented the highest CNVR coverage of a chromosome (5.62%) with 14 CNVR. The total number of CNVR inferred in the autosomal chromosomes covered 58,928,942 bp, which corresponds to 2.60% of the pig autosomal genome size. Most of the CNVR presented low frequency in the population (Figure 2). However, 52 CNVR were considered the most common in the population, shared by at least 1% (16 animals) of all genotyped individuals (Table 2).

**TABLE 1:** Summary of the copy number variation regions (CNVRs) detected in autosomal chromosomes in pigs.

Chr^a^	Chr length (bp)	Number of CNVRs	CNVR length (bp)	Coverage (%)^b^
chr1	274,330,532	25	3,803,364	1.39
chr2	151,935,994	26	5,410,473	3.56
chr3	132,848,913	11	2,826,150	2.13
chr4	130,910,915	11	2,692,015	2.06
chr5	104,526,007	16	4,931,129	4.72
chr6	170,843,587	6	2,055,133	1.20
chr7	121,844,099	9	1,812,412	1.49
chr8	138,966,237	8	1,165,734	0.84
chr9	139,512,083	13	1,729,061	1.24
chr10	69,359,453	7	1,224,889	1.77
chr11	79,169,978	14	4,452,633	5.62
chr12	61,602,749	9	3,044,686	4.94
chr13	208,334,590	21	5,224,056	2.51
chr14	141,755,446	20	6,463,588	4.56
chr15	140,412,725	19	5,833,526	4.15
chr16	79,944,280	12	4,002,490	5.01
chr17	63,494,081	5	1,599,001	2.52
chr18	55,982,971	4	658,602	1.18
Total	2,265,774,640	236	58,928,942	2.60

^a^ Chromosome.

^b^ Percentage of the chromosome covered by the copy number variation regions.

**TABLE 2:** Autosomal copy number regions (CNVRs) identified in at least 1% of the population of crossbred (Large White × Landrace) sows.

Chr	Start (bp)	End (bp)	Type	Number of individuals
1	20,559,479	21,017,894	Deletion	23
1	26,407,492	26,757,112	Deletion	24
2	1111	632,707	Deletion	202
2	6,179,112	6,495,647	Deletion	34
2	38,779,149	39,302,035	Deletion	22
2	129,799,406	129,981,864	Deletion	70
2	130,241,255	130,487,175	Deletion	58
3	60,993	905,589	Deletion	28
3	17,333,664	17,509,970	Deletion	47
3	69,789,999	70,298,496	Deletion	17
4	1,155,293	1,388,468	Deletion	61
4	80,721,007	80,767,798	Deletion	22
4	126,897,791	127,521,733	Deletion	28
5	64,630,743	65,275,886	Deletion	32
5	77,179,466	77,352,621	Deletion	28
6	1,033,257	1,281,226	Deletion	101
6	132,124,953	132,543,418	Mixed	139
7	50,084,357	50,518,151	Deletion	16
8	114,376,195	114,407,527	Deletion	22
8	123,960,362	124,128,258	Deletion	95
8	137,114,772	137,134,554	Deletion	23
9	143,288,947	143,605,209	Deletion	78
10	48,034,226	48,281,381	Deletion	66
11	46,563,594	46,642,716	Deletion	90
11	59,735,869	61,470,768	Mixed	203
11	64,479,055	65,142,186	Deletion	45
11	67,375,641	67,671,825	Deletion	20
12	60,100	1,998,701	Mixed	93
13	102,306,232	102,458,997	Deletion	31
13	194,665,680	196,342,442	Deletion	108
13	200,765,190	201,339,708	Deletion	36
14	16,993,535	17,333,398	Deletion	63
14	38,219,290	38,645,949	Deletion	21
14	74,176,726	74,990,114	Deletion	24
14	104,441,890	105,084,009	Mixed	54
14	136,444,819	137,178,653	Deletion	64
14	140,199,680	140,419,493	Deletion	18
14	151,966,127	153,374,972	Deletion	80
15	9,519,813	10,711,345	Deletion	245
15	12,597,728	12,844,713	Deletion	80
15	24,371,978	24,804,276	Deletion	63
15	30,831,468	31,122,672	Deletion	19
15	48,845,490	49,845,075	Deletion	47
15	126,439,963	127,295,381	Deletion	45
15	134,842,540	134,994,861	Deletion	62
16	2,294,773	2,931,783	Deletion	20
16	10,588,133	11,083,680	Deletion	43
16	14,404,612	14,706,916	Deletion	26
16	85,407,012	86,024,383	Deletion	22
17	2,202,762	2,730,970	Deletion	184
17	4,055,697	4,468,083	Deletion	22
18	38,984,087	39,185,391	Deletion	32

### 3.2 CNV and CNVR Detection in the X Chromosome

A total of 261 animals had at least one CNV detected in their X chromosome. Initially, 280 CNV were detected in the X chromosome. After quality control, 188 CNV (55 deletion and 133 duplication) for 173 animals were kept for subsequent analyses. A maximum of 2 CNV on the X chromosome were observed per individual. The length of the CNV located on chromosome X ranged from 14,613 to 788,523 bp, with an average (SD) length of 52,270.06 (74,726.51) bp. After merging the 188 CNV with at least 1 bp overlap, 16 CNVR were observed. However, four of them were removed due to being present in a single sample. Therefore, 12 CNVR (Table 3), including four deletion‐type CNVR and 8 duplications, covering 2,413,025 bp (1.92%) of the X chromosome remained for further analyses.

**TABLE 3:** Copy number variation regions (CNVRs) detected on the X chromosome in crossbred pigs.

CNVR	Start (bp)	End (bp)	Type	*N* ^a^
CNVRX1	2,409,197	2,626,938	Deletion	44
CNVRX2	4,405,540	4,666,913	Deletion	5
CNVRX3	6,722,207	6,798,065	Deletion	2
CNVRX4	8,887,538	9,153,402	Duplication	6
CNVRX5	13,940,093	14,047,721	Duplication	3
CNVRX6	46,678,999	46,814,346	Deletion	3
CNVRX7	101,926,902	101,973,628	Duplication	97
CNVRX8	107,241,584	107,333,960	Duplication	4
CNVRX9	109,697,037	110,485,560	Duplication	2
CNVRX10	120,643,237	120,801,309	Duplication	3
CNVRX11	132,300,938	132,440,533	Duplication	10
CNVRX12	135,914,828	136,038,750	Duplication	5

^a^ Number of animals with the CNVR.

### 3.3 Associations Between CNVR and Physiological and Anatomical Indicators of HS Response in Lactating Sows

A total of 54 CNVR, including 52 on autosomes and 2 on the X chromosome, shared by at least 1% of the population, were used to perform the association analyses. The λ values (genomic inflation factor) ranged from 0.73 to 1.57 prior to the adjustment of the *p*‐values and from 0.96 to 1.02 after the *p*‐value adjustments. These analyses led to the identification of 3 CNVR significantly associated with T_ES_, and 1 CNVR (CNVR168) significantly associated with T_Vall_, T_V8h_ and HD (Table 4).

**TABLE 4:** Copy number variation regions (CNVRs) associated with indicators of heat stress response in pigs.

Trait^a^	CNVR^b^	CHR	Start	End	Type	*b* ^c^	SE	*p* adjusted^d^
T_ES_	CNVR152	chr15	24,371,978	24,804,276	Deletion	1.286563	0.347736	0.0000
	CNVR174	chr17	2,202,762	2,730,970	Deletion	0.425037	0.19818	0.0002
	CNVR72	chr6	132,124,953	132,543,418	Mixed	0.48327	0.228122	0.0002
T_Vall_	CNVR168	chr16	14,404,612	14,706,916	Deletion	−0.24152	0.092293	0.0002
T_V8h_	CNVR168	chr16	14,404,612	14,706,916	Deletion	−0.33116	0.110379	0.0007
HD	CNVR168	chr16	14,404,612	14,706,916	Deletion	−0.40662	0.143341	0.0004

*Note*: significant at the 0.05 level.

^a^ HD, hair density; T_ES_, ear skin surface temperature; T_V8h_, vaginal temperature measured on the first day at 8:00 h; T_Val_, vaginal temperatures included all measures (every 10 min) of vaginal temperatures for four days.

^b^ Chr: chromosome.

^c^ Effect size.

Eleven annotated genes overlapped with the significantly associated CNVR with T_ES_ (*ENSSSCG00000031239*, *STEAP3*, *EN1*, *MARCO*, *C1QL2*, *ENSSSCG00000055496*, *SGCZ*, *ssc‐mir‐383*, *ENSSSCG00000053778*, *ENSSSCG00000059804* and *ENSSSCG00000060648*), there were no genes identified in overlap with the CNVR168 associated with T_Vall_, T_V8h_ and HD. The genes annotated within these significant CNVR were subjected to functional genomic analyses; however, there were no GO terms identified.

## 4 Discussion

### 4.1 Copy Number Variation and CNVR

The number of autosomal CNVR identified in this study is in the range reported by previous studies in pigs. Using the Illumina Porcine SNP60 BeadChip (Illumina Inc., San Diego, CA, USA) for CNVR detection, 170 CNVR (7 deletions, 161 duplications and 2 mixed type) were identified in 293 Large White pigs (Schiavo et al. 2014); 249 CNVR (70 deletions, 43 duplications and 136 mixed) were detected in 585 crossbred (Large White × Minzhu) pigs (Wang et al. 2013); and 348 CNVR (243 deletions, 88 duplications and 17 mixed) were observed in 302 animals representing ten Chinese pig breeds (Wang et al. 2014). Using the Illumina Porcine SNP50 BeadChip (Illumina Inc., San Diego, CA, USA), Qiu et al. (2021) identified 953 CNVR (376 deletions, 388 duplications and 189 mixed type) in 5928 Duroc pigs. Stafuzza et al. (2019) identified 425 CNVR (342 deletions, 19 duplications and 64 mixed type) in 3520 Duroc pigs genotyped using the Porcine SNP80 BeadChip (Illumina Inc., San Diego, CA, USA). Also using the Illumina Porcine SNP80 BeadChip, Wang et al. (2020) detected 312 CNVR in 1199 Large White pigs (184 deletions, 66 duplications and 62 mixed type).

While CNV on the X chromosome has been thoroughly investigated in humans and reported to impact fertility in both males and females (Knauff et al. 2011; Chianese et al. 2014; Huang et al. 2019; Yatsenko et al. 2019), there is a lack of studies reporting CNV on the X chromosome in livestock. Kijas et al. (2011) utilised a Comparative Genomic Hybridization (CGH) array containing approximately 385,000 oligonucleotide probes and identified 7 CNVR on the X chromosome in a sample of 10 cattle, including three Angus, six Brahman, and one composite animal. Jiang et al. (2013) observed 9 CNVR (3 deletion, 5 duplication and 1 mixed type) in 96 Holstein cattle genotyped with the Illumina High‐Density BovineSNP beadChip (Illumina Inc., San Diego, CA, USA) containing 777,692 SNPs. Zhu et al. (2020) reported six, four and 22 CNVR located on the X chromosome in 20 large‐tailed Han, 15 Altay and 17 Tibetan sheep, respectively. Studying local Chinese pig breeds, Xie et al. (2016) reported 22 CNVR (3 deletion and 19 duplications) in 98 Xiang pigs and 22 Kele pigs genotyped with the Illumina PorcineSNP60K BeadChip (Illumina Inc., San Diego, CA, USA). In this study, we observed 12 CNVR on the X chromosome, which is consistent with previously reported findings using similar SNP density panels.

The variation in the number of identified CNVR in different studies can be attributed to the population‐specific characteristics associated with CNV (Chen et al. 2012; Hou et al. 2012; Pierce et al. 2018). These variations are sources of both interindividual differences and variations between breeds (Pierce et al. 2018). Furthermore, differences in the genotyping strategies and platforms, methodologies and software used for the analyses, quality control criteria, SNP panel density and variations in sample size can contribute to the observed differences in the identification of CNV and CNVR among pig populations (Redon et al. 2006; Duan et al. 2013; Zhao et al. 2013). The proportion of the autosomal genome covered by CNVR (2.60%) observed in this study aligns with values reported in the literature on pigs, which ranged from 0.36% to 10.90% (Wang et al. 2013, 2014, 2020; Stafuzza et al. 2019; Qiu et al. 2021). The observed variation in genome coverage across studies can be explained by the different SNP panels used. The choice of SNP panels has the potential to impact the sensitivity of CNV detection, such as the sensitivity in detecting small CNV (Bernardini et al. 2010).

We also compared our results with those of previous studies on CNVR in pigs that used similar genotyping platforms, the same software to identify the CNV, and were based on the version Sscrofa11.1 of the pig reference genome. A total of 140 CNVR identified in this study overlapped with CNVR reported in previous studies (Table S2). More specifically, 132 CNVR overlapped with those reported by Stafuzza et al. (2019) in Duroc pigs, 64 CNVR overlapped with those identified by Wang et al. (2020) in Large White pigs and 72 CNVR overlapped with those reported by Qiu et al. (2021) in Duroc pigs. According to Paudel et al. (2013), the majority of CNVR were already segregating among wild boars before domestication and domestication did not lead to a change in CNVR among breeds, resulting in many CNVR being shared among different populations of pigs.

### 4.2 CNVR Associated With Physiological and Anatomical Indicators of HS Response in Lactating Sows

The variance components and genetic parameters for the traits evaluated in this study have been previously reported by Freitas et al. (2023). Skin temperatures (i.e., T_ES_, T_SS_, T_RS_ and T_TS_), RR, and PS exhibited low heritability estimates, ranging from 0.04 to 0.06. Vaginal temperatures (i.e., T_Vall_, T_V4days_, T_V8h_ T_V12h_, T_V16h_ and T_V20h_) are moderately heritable, with estimates ranging from 0.15 to 0.29. Using the same dataset, Hui et al. (2023) reported estimates ranging from 0.14 to 0.20 throughout the day when studying vaginal temperature as longitudinal traits using a random regression model. Anatomical traits (i.e., BCS_cal_, BCS_vis_, HD, BS, EA and EL) showed moderate to high heritability, ranging from 0.25 to 0.40 (Freitas et al. 2023).

In a previous study, Oliveira et al. (2024) performed a GWAS using the same dataset, with genotypes imputed to whole‐genome sequence data. We observed that six CNVR identified in this study overlapped with genomic regions found to be significant by Oliveira et al. (2024) for T_V8h_, T_RS_, T_SS_ and HD. Genomic structural variations, such as CNV, may contribute to additive variance and consequently to trait heritability (Nagao 2015). They also influence gene expression level (Henrichsen et al. 2009) and consequently impact the phenotypic variability of complex traits. The investigation of the functions of these genomic alterations provides valuable insights into understanding inheritance patterns and the variability of phenotypes within populations.

We identified 4 CNVR significantly associated with T_ES_, T_Vall_, T_V8h_ and HD. These regions overlap with 11 genes, most of which are involved in immune response. Notably, pigs under HS conditions experience increased body temperature (Cervantes et al. 2016; Yu et al. 2020), which generally promotes the activation of immune cells, while reduced temperatures inhibit these processes (Coiffard et al. 2021). CNVR152, which was a deletion region associated with T_ES_, includes the genes *STEAP3* (STEAP3 metalloreductase) and *MARCO* (Macrophage receptor with collagenous structure). *STEAP3* is involved in the maintenance of iron homeostasis and modulation of immune responses in macrophages. *MARCO* acts in the innate immune system by binding and removing bacteria and has been implicated in actin cytoskeleton rearrangements (Komine et al. 2013). It has also been shown that *MARCO* modulates the induction of T cell responses, indicating its function in adaptive immunity (Komine et al. 2013). We observed that sows carrying the CNVR152 deletion exhibited significantly higher T_ES_, with an estimated effect of 1.28 ± 0.34. This suggests that the deletion may impair immune regulatory pathways, potentially altering inflammatory signalling or vascular responses under heat stress conditions. Such disruption could lead to reduced vasoconstriction or impaired heat dissipation, resulting in elevated peripheral temperature. These findings are consistent with our hypothesis that immune function is closely linked to thermoregulatory responses during heat stress.

### 4.3 Implications and Limitations

The observations of this study offer valuable insights into the impact of CNV in pigs. Notably, several genes involved in immune and stress responses were identified within the most frequently occurring CNVR in the population. This implies a potentially important role of these CNVR in adaptation. The CNVR may contribute to more resilient animals to stress factors such as diseases and HS.

The number of animals used for CNV detection in this study is relatively small; while a higher number of samples is desirable to reduce the probability of false‐positive CNV. A larger dataset would also improve the power to detect associations between the CNVR and the traits evaluated. Moreover, this study utilised a medium‐density SNP array to obtain genotypes, which may not be the most optimal approach for CNV calling, especially for detecting shorter CNV, which tend to have reduced accuracy. A higher marker density panel or whole‐genome sequence data could enhance the detection of short CNV and potentially reduce the number of false‐positive CNV (Bernardini et al. 2010).

## 5 Conclusions

This study aimed to investigate CNV and CNVR in the genome of crossbred sows (Large White × Landrace) and their associations with physiological and anatomical indicators of HS response during lactation. Several CNV and CNVR were identified, with four CNVR showing significant associations with indicators of HS response. These findings suggest that the identified CNVR may be involved in biological processes related to adaptation and resilience to HS. The CNVR significantly associated with HS response indicators contain genes related to immune functions, which are essential processes in the HS response and may influence the adaptation and resilience to HS.

## Author Contributions

L.F.O., R.V., J.S.J. and L.F.B. conceived and designed the research. L.F.O. conducted the analyses. L.F.B. and A.C.A. helped with the data analyses. R.V. and L.F.B. contributed to the study supervision, interpretation and discussion of the results. J.S.J., Y.H. and L.F.B. generated the datasets used. L.F.O. wrote the first version of the manuscript. L.F.B., R.V., J.S.J., Y.H. and A.C.A. edited the manuscript. All authors read and approved the final version of the manuscript.

## Ethics Statement

The Purdue University Animal Care and Use Committee approved all procedures involving pigs (Protocol #1912001990) during data collection. Animal husbandry and use protocols were based upon the Guide for the Care and Use of Agricultural Animals in Research and Teaching (Federation of Animal Science Societies, 2020). All methods have been reported in accordance with ARRIVE (Animal Research: Reporting of In Vivo Experiments) guidelines.

## Conflicts of Interest

Y.H. was employed by Smithfield Foods. The remaining authors declare no conflicts of interest.

## Supporting information


**Table S1:** Copy number regions (CNVRs) identified in crossbred (Large White × Landrace) sows.

**Table S2:** CNVR overlapping with previous reported in the literature.

## Data Availability

The dataset can be made available for research purposes by contacting Dr. Luiz Brito (britol@purdue.edu).
